# Assessing hand hygiene knowledge, attitudes, and behaviors among Guatemalan primary school students in the context of the COVID-19 pandemic

**DOI:** 10.1186/s12889-023-17168-4

**Published:** 2023-11-16

**Authors:** Michelle M Pieters, Natalie Fahsen, Ramiro Quezada, Caroline Pratt, Christina Craig, Kelsey McDavid, Denisse Vega Ocasio, Christiana Hug, Celia Cordón-Rosales, Matthew J. Lozier

**Affiliations:** 1 Center for Health Studies, Universidad del Valle de Guatemala, Guatemala City, Guatemala; 2 Waterborne Disease Prevention Branch, Division of Foodborne, Waterborne, and Environmental Diseases, Centers for Disease Control and Prevention, Atlanta, GA USA; 3 Epidemic Intelligence Service, Centers for Disease Control and Prevention, Atlanta, GA USA

**Keywords:** Hand hygiene, Knowledge, Attitudes, Practices, Hard dirtiness, Hand hygiene observations, Hand hygiene behavior, COVID-19

## Abstract

**Background:**

Hand hygiene (HH) is an important practice that prevents transmission of infectious diseases, such as COVID-19. However, in resource-limited areas, where water and soap are not always available, it can be difficult to practice HH correctly and at appropriate moments. The purpose of this study was to assess HH knowledge and behaviors among students from six elementary schools in Quetzaltenango, Guatemala to identify gaps that could later inform interventions to improve HH.

**Methods:**

We conducted knowledge, attitude, and practices (KAP) surveys among primary school students during the COVID-19 pandemic in July 2022. We also observed students’ HH practices at three different moments during the day, making note of the use of the HH station and materials, duration of handwashing, presence of a HH assistant, and the students’ sex. We also used the Quantitative Personal Hygiene Assessment Tool (qPHAT), to measure hand dirtiness before eating, after restroom use, and upon arriving to school.

**Results:**

We surveyed 109 students across six schools. Mean scores were 4 out of 5 for knowledge, 8 out of 8 for attitudes, and 6 out of 7 for HH practices. Most students identified “before eating” as a critical moment for HH (68.8%), fewer identified “after restroom use” (31.2%), and no students mentioned HH being necessary “after coughing or sneezing”. We observed 326 HH opportunities of which 51.2% performed correct HH (used water and soap for at least 20 s or used alcohol-based hand rub, where materials were available). We collected 82 qPHAT hand swabs. A Kruskal Wallis test revealed a significant difference in hand dirtiness between entering the school and after restroom use (p = 0.017), but no significant difference before eating and after entering the school (p = 0.6988).

**Conclusions:**

The results from the KAP survey show high scores, however correct identification of key moments for HH was relatively uncommon, especially after restroom use and after coughing or sneezing. Additionally, half of HH opportunities observed had correct HH practices and on average, hands were dirtiest when arriving at school. These findings will inform interventions to improve HH practices and behaviors, which will be evaluated with follow-up data collection.

## Background

School children are susceptible to infectious diarrheal diseases and respiratory illnesses, such as COVID-19, due to large amount of contact with other children at school and their underdeveloped immune systems [1]. These infections impact children´s health and cause missed educational opportunities, which has a negative impact on educational outcomes [2]. Studies have shown that hand hygiene (HH), defined as washing hands with soap and water or using alcohol-based hand rub (ABHR) with 60–95% alcohol, prevents the spread of respiratory and diarrheal infections among students [3].

COVID-19 was declared a pandemic by the World Health Organization on March 11, 2020, which prompted recommendations to increase mitigation efforts. These recommendations included avoiding crowds, wearing face masks, and cleaning hands frequently with ABHR or soap and water [4]. In Guatemala, schools were required by the Ministry of Education (MINEDUC) and the Ministry of Health (MSPAS, its Spanish acronym) to have at least one HH station per classroom, either with soap and water or ABHR [5, 6]. However, despite the well-known benefits of HH practice, studies have shown that primary school students do not practice proper HH. In a cross-sectional study carried out in Ethiopia, it was found that only a third of primary school students practiced proper HH [7]. Similarly, studies from Malaysia and Ghana have evidenced that only around 20–30% of students practice HH correctly, even though their knowledge of the importance of HH is high [8, 9].

To date, little is known about knowledge, attitudes, and behaviors of HH among elementary school children in Guatemala. The literature gap underscores the importance of conducting research in this area, as understanding these aspects can serve as a foundation for developing programs aimed at improving HH among schoolchildren. Therefore, the aim of this study was to examine and establish a baseline of Guatemalan primary school students’ HH knowledge and behaviors through a knowledge, attitudes and practices (KAP) survey, direct HH observations, and hand dirtiness evaluations.

## Methods

### Study design and setting

**Fig. 1:**
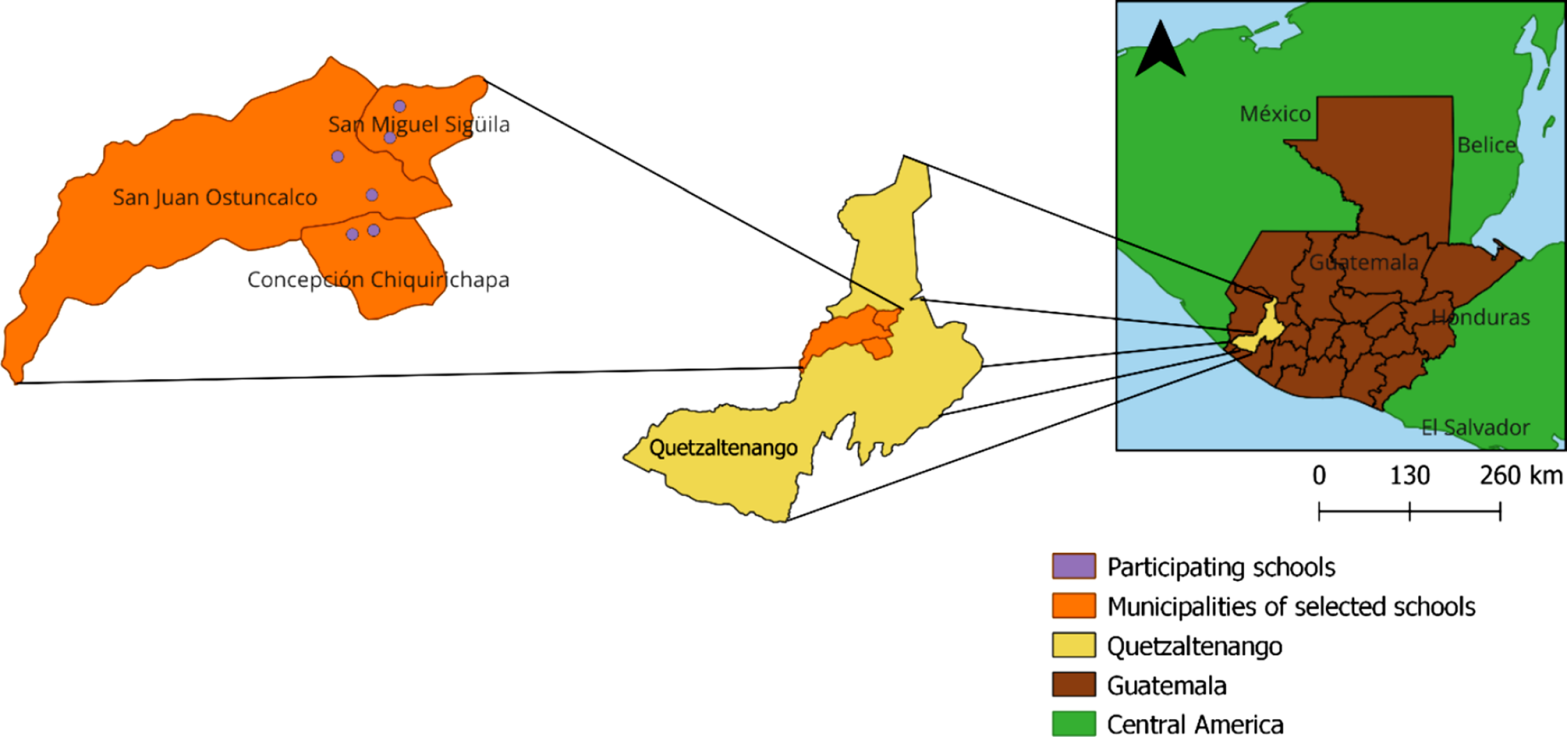
Map of study location

This research used a cross-sectional study design, incorporating knowledge, attitudes and practices (KAP) surveys, direct hand hygiene observations, and a hand dirtiness evaluation, to comprehensively assess HH knowledge, attitudes and behaviors among public primary school students. The study focused on six primary schools in three municipalities in the department of Quetzaltenango, Guatemala. Two schools were selected from the municipalities of San Miguel Sigüilá, San Juan Ostuncalco, and Concepción Chiquirichapa, respectively (Fig. 1). Two schools are in urban areas and four schools are in rural areas. All schools were gender-mixed, except for one, which is all-boys school. Schools were selected by convenience and according to a priori selection criteria (had in-person classes and were willing to participate in the study).

### Study participants and eligibility criteria

All students attending classes in the selected schools were a source population. KAP surveys were administered to students from 3rd to 6th grades, as children had to be over eight years old to participate to guarantee accurate comprehension of the survey questions. Observations were conducted throughout the school day; therefore, any student could be selected for observation. Similarly, for the hand dirtiness evaluation, any student walking into school, using the restroom, or going to eat was conveniently selected. Students participating in KAP surveys and hand dirtiness evaluation had to provide their verbal assent to participate.

### Data collection

Data were collected between April 20th and May 11th, 2022. KAP surveys were pilot tested for general understanding with students and modified accordingly before data collection started. The surveys were conducted in Spanish during the school day by two enumerators, and they recorded responses using REDCap electronic data capture tool on a tablet [10]. Answer options were not shown or read aloud to participants. Each survey had 30 questions and took approximately 10 min to complete.

We observed hand hygiene practices of students of all ages present in the school. Observations of HH practices were carried out at three different moments during the day: as students entered the school, after they used the restroom, and before eating. Enumerators stood in an unobtrusive area near the entrance of the school, close to bathrooms, and outside the classrooms to observe. Students were observed for a predetermined amount of time (e.g., for the duration of recess at each school), or until 20 observations were carried out, whichever occurred first. Enumerators observed: (1) if students attempted HH, (2) the type of HH attempted (handwashing with water only, handwashing with soap and water, or using ABHR), (3) the handwashing duration (< 20 s or ≥ 20 s), (4) the student’s sex, (5) whether there was a HH assistant present and (6) what HH materials were present. A HH assistant was defined as anyone who was actively telling or showing the students how to wash or clean their hands. Observations were only carried out if a HH station was available during the selected moments. Observation data were collected on paper, and later transferred to REDCap. Students were aware that a team was observing them but were not told what was being observed.

**Fig. 2:**
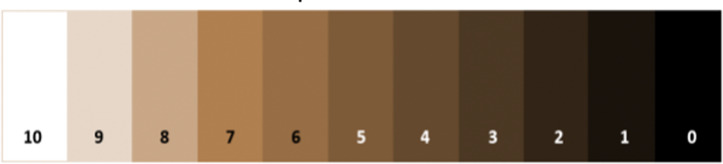
qPHAT 11-point color scale

The Quantitative Personal Hygiene Assessment Tool (qPHAT) was used to assess hand dirtiness from students [11]. It involves tracing the palm and fingertips of the participant’s hand with a pre-moistened sterile saline gauze pad and comparing the darkest half-square inch area of the gauze pad against the qPHAT color scale. The qPHAT uses a scale from 0 to 10, where 0 indicates the most visible dirt possible on a hand and 10 indicates the absence of any visible debris or dirt (Fig. 2) [11]. Hand swabs were collected at three different moments during the day: (1) as students entered the school, (2) after restroom use whether or not they practiced HH, and (3) before they washed their hands before eating. After the sample was collected, the score of the swab on the qPHAT scale was determined and agreed upon by two enumerators. Scores were entered into REDCap along with student´s demographic information (age, grade, and sex).

### Sample size and sampling technique

For the KAP surveys we estimated we needed 144 surveys (24 per school) to be able to detect a significant difference between pre- and post-intervention scores with 90% confidence. KAP surveys were carried out with conveniently selected students to avoid disrupting class time as much as possible (i.e., student had finished their classwork and/or was caught up with class).

For HH observations, we determined a sample size based on the duration of observation period (however long recess lasted) or a maximum of 20 students observed per period. Therefore, we calculated a maximum amount of 360 observations in total (60 observations per school – 20 observations maximum per observation period).

**Fig. 3:**
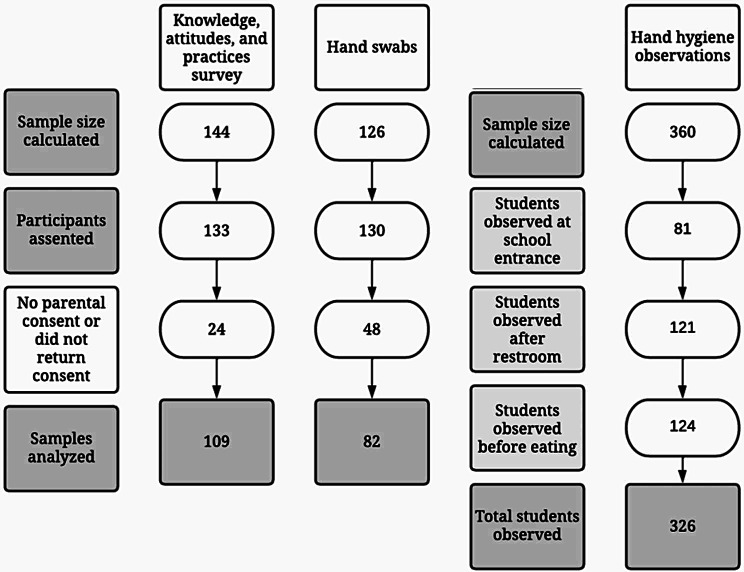
Sampling flowchart by study activity

For the hand dirtiness evaluation, we estimated we needed 120 swabs (20 swabs per school) to be able to detect a significant difference between pre and post intervention scores with 95% confidence. Therefore, we collected seven hand swabs per HH moment per school for 21 hand swabs per school and 126 hand swabs in total. Students were selected by convenience based on who entered school, used the restroom during the school day and went out for recess. See Fig. 3 for description of sampling flowchart by study activity.

### Statistical analysis and variables of interest

Descriptive statistics of all three evaluation tools were generated with STATA version 17 [12]. Participants data was analyzed only if there were no missing data on the variables of interest. For the KAP surveys, a scoring system was developed a priori and applied after all surveys were collected. Each correct response was given one point, and all points were added up to create a scale ranging from 0 to 5 for knowledge, 0 to 8 for attitudes, and 0 to 7 for practices. Scores and correct responses to questions are presented as frequencies and percentages. For the observations, we calculated correct hand hygiene practices – defined as using water and soap for at least 20 s or using ABHR – only where appropriate materials were available. We also performed chi-square tests to assess associations between sex and presence of a hand hygiene assistant on correct HH practice. For the hand dirtiness evaluations, we conducted a Kruskal Wallis test to assess the difference in hand dirtiness at different moments during the day and the association between scores and school location (urban or rural). A significance level of 0.05 was used for the chi-square and Kruskal Wallis tests.

## Results

Overall, 109 (82%) of 133 KAP surveys were included in the data analysis and 82 (63%) of 130 hand swabs were included. All 326 HH observations were included in the analysis.

### Demographic data

Between 12 and 23 students participated from each school. Among these 109 KAP survey respondents, 56 (51.4%) were male, the mean age (± standard deviation) of the participants was 10 ± 1 years, and 4th grade was represented the most (32.1%, n = 35) (Table 1). Of the 326-hand hygiene observations conducted, 176 (54.2%) students were male. Half (50.0%, n = 41) of students that participated in the hand swabs were male, and the mean age of was 10 ± 1.

**Table 1:** Demographic characteristics of participants in KAP survey, hand swabs, and hand hygiene observations

	KAP participants [n = 109]	Hand hygiene observations [n = 326]	Hand swabs participants [n = 82]
	n (%)	n (%)	n (%)
**Sex+**			
*Male*	56 (51.4)	176 (54.2)	41 (50.0)
*Female*	53 (48.6)	149 (45.9)	41 (50.0)
Age*			
*8*	10 (9.2)	-	7 (8.5)
*9*	24 (22.0)	-	19 (23.2)
*10*	36 (33.0)	-	26 (31.7)
*11*	15 (13.8)	-	19 (23.2)
*12*	19 (17.4)	-	7 (8.5)
*13*	5 (4.6)	-	3 (3.7)
*14*	0 (0.0)	-	0 (0.0)
*15*	0 (0.0)	-	1 (1.2)
Grade*			
*3rd*	27 (24.8)	-	18 (22.0)
*4th*	35 (32.1)	-	32 (39.0)
*5th*	27 (24.8)	-	14 (17.0)
*6th*	20 (18.4)	-	18 (22.0)

+One missing value in observations

*Age and grade were not collected when doing hand hygiene observations

### Knowledge, attitudes, and practices survey

#### Knowledge

Overall mean knowledge scores were high as 73% (n = 80) scored four or more out of five (Table 2). Just over half (53.2%, n = 58) of participants knew that hands should be washed for 20 s or more. More than one third (43.1%, n = 47) of KAP survey participants correctly identified that handwashing is important to prevent spreading germs, and 56.0% (n = 61) reported to avoid getting sick. 78% of participants (n = 85) correctly answered that water and soap should be used to clean hands that are visibly dirty. When asked about the critical moments for HH, 75 (68.8%) students correctly stated that people should wash their hands “before eating”, 34 (31.2%) stated “after using the restroom”, and no student mentioned it being important to conduct HH “after coughing or sneezing”.

**Table 2:** Distribution of responses to the knowledge questions

Knowledge Questions	n (%) [n = 109]
**Overall knowledge score**	
*5*	29 (26.6)
*4*	51 (46.8)
*3*	23 (21.1)
*2*	6 (5.5)
*1*	0 (0)
**For how many seconds do you think you should wash your hands?**	
20 s or more*	58 (53.2)
*Less than 20 s*	35 (32.1)
*Don’t know*	15 (13.8)
*Did not respond*	1 (0.9)
What materials are needed for hand hygiene?**	
Water and soap OR alcohol based hand rub*+	103 (94.5)
*Towel*	25 (22.9
*Paper*	1 (0.9)
*Other*	3 (2.8)
*Just water*	3 (2.8)
*Don’t know*	3 (2.8)
Why is hand hygiene important?**	
To avoid getting sick*	61 (56.0)
To stop germs from spreading*	47 (43.1)
*Don’t know*	11 (10.1)
*Other*	8 (7.3)
To remove visible dirt*	5 (4.6)
**If your hands are visibly dirty, what materials should you use to wash them?**	
Water and soap*	85 (78.0)
*Just water*	13 (11.9)
*Alcohol based hand rub*	8 (7.3)
*Don’t know*	2 (1.8)
*Other*	1 (0.9)
When should you wash your hands?**	
Before eating*	75 (68.8)
After using the restroom*	34 (31.2)
After eating*	17 (15.6)
After touching something that is dirty*	16 (14.7)
*Don’t know*	15 (13.8)
After playing outside*	12 (11.0)
*Other*	7 (6.4)
*Did not respond*	2 (1.8)
After coughing or sneezing*	0 (0.0)

*Correct answer

**Answer percentages add up to more than 100 because participants could give more than one answer

+For scoring purposes, we included water and soap or ABHR under the same answer option

#### Attitudes

Two-thirds (66.1%; n = 72) of students scored eight out of eight, followed by 22.9% (n = 25) that scored 7 points, and 10.1% (n = 11) that scored 6 points. One student scored a five, and no student scored four or less points.

Across all questions, the majority of students demonstrated positive attitudes (answered “yes”) towards HH. For example, 95.4% (n = 104) answered that they believe handwashing is important to prevent diseases (Table 3). When asked about perceived attitudes towards HH from friends and family responses were also positive. A higher proportion of participants indicated that they like using soap and water to wash their hands compared to ABHR to clean their hands (109 [100%] vs. 95 [89.0%], respectively).

**Table 3:** Student attitudes toward hand hygiene

Attitude	Yes [n = 109] n (%)
*Personally like using soap and water to wash hands*	109 (100.0)
*Friends and family wash their hands with soap and water*	106 (97.2)
*Easy to wash hands at home*	107 (98.2)
*Hand washing is important to prevent diseases*	104 (95.4)
*Easy to wash hands at school*	104 (95.4)
*Friends and family think handwashing is important*	100 (91.7)
*Personally like using alcohol-based hand rub to clean hands*	97 (89.0)
*Friends and family use alcohol-based hand rub*	95 (87.2)

#### Practices

Out of 7 possible points, 44.0% (n = 48) of students had a perfect score, 33.9% (n = 37) of students scored a six, 13.8% (n = 15) scored a five, and the remainder of students scored between four and one points (8.3%, n = 9).

Almost all students (89.9%, n = 98) reported washing their hands that day at some point prior to participating in the survey (Table 4). Of those students, 90 (91.8%) reported using an appropriate hand hygiene method (either soap and water or ABHR) while 7 (7.1%) reported using water only. Almost all students mentioned that they always practice hand hygiene at home (96.3%, n = 105) and use either soap and water or ABHR (97.2%, n = 106). Similarly, students also reported they always wash their hands at school (89.9%, n = 98). When asked how long they take to wash their hands, 75 (68.8%) students said they take 20 s or more. When assessed about their perceived HH practices at different moments during the day, 53 (48.6%) said they wash their hands “after using the restroom” and 67 (61.5%) mentioned washing their hands “before eating”. No student mentioned washing hands “after coughing or sneezing”.

**Table 4:** Distribution of responses for practice questions

Practice Questions	n (%) [n = 109]
**Have you washed your hands today?**	
Yes*	98 (89.9)
**What did you use to wash your hands today?**	**[n = 98]**
Soap and water or alcohol based hand rub*	90 (91.8)
*Water only*	7 (7.1)
*Other*	1 (1.0)
**Do you wash your hands at home?**	
Yes, always*	105 (96.3)
*Sometimes*	4 (3.7)
**What do you use at home to wash your hands?+	
Soap and water or alcohol based hand rub*	106 (97.2)
*Towel*	7 (6.4)
*Water only*	3 (2.8)
*Other*	1 (0.9)
*Don’t know*	1 (0.9)
**Do you wash your hands at school?**	
Yes, always*	98 (89.9)
*Sometimes*	11 (10.1)
**When you wash your hands, how long do you take?**	
20 s or more*	75 (68.8)
*Less than 20 s*	26 (23.85)
*Don’t know*	8 (7.34)
When do you practice hand hygiene?**	
Before eating*	67 (61.5)
After using the restroom*	53 (48.6)
After playing outside*	19 (17.4)
After touching something that is dirty*	14 (12.8)
After eating*	13 (11.9)
*Don’t know*	10 (9.2)
*Other*	6 (5.5)
*Did not respond*	4 (3.7)
After coughing or sneezing*	0 (0.0)

*Correct practice

**Answer percentages add up to more than 100 because participants could give more than one answer

+for scoring purposes, we included water and soap or ABHR under the same answer option

### HH observations

Of a total of 326 observations, 81 observations were carried out at school entrances, 121 after restroom use, and 124 before eating. A HH assistant was present in 79 (97.5%), 21(17.5%), and 29 (23.4%) of observations at entrance, after restroom use, and before eating, respectively. Out of the students observed 54.2% (n = 176) were male.

**Fig. 4:**
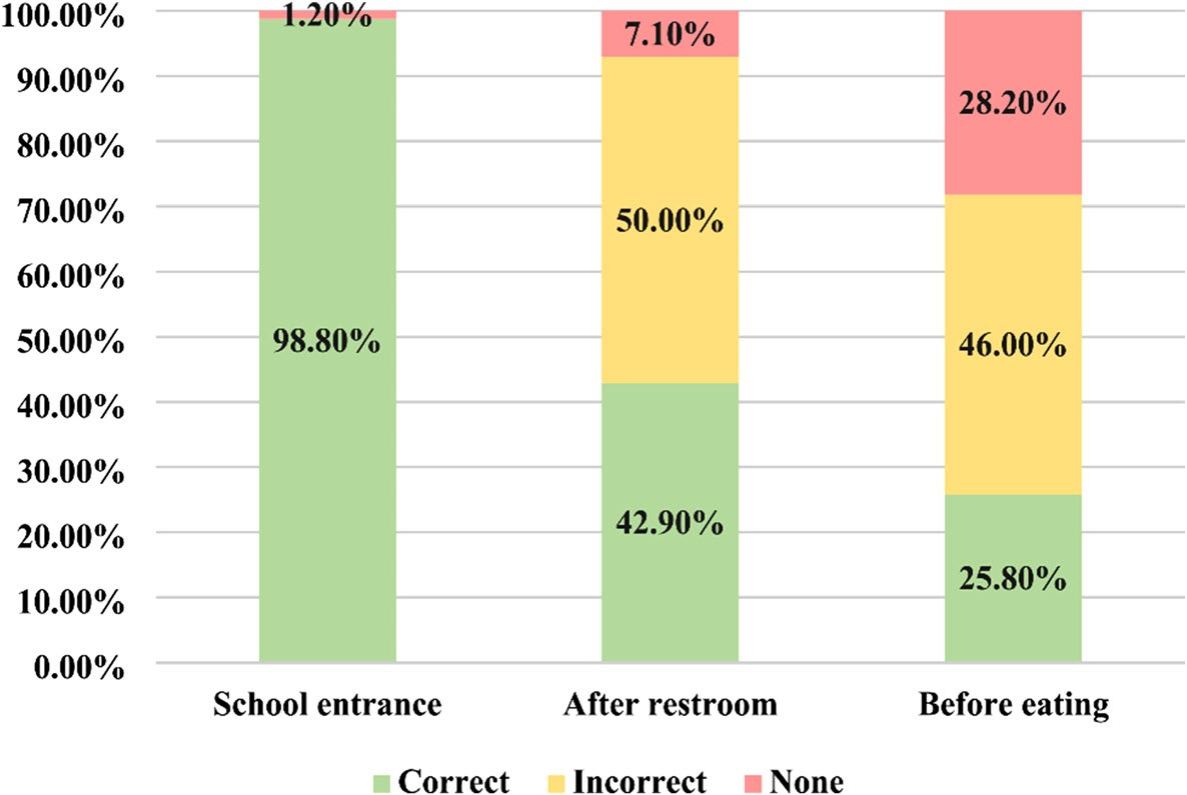
Observed hand hygiene practices

Among the total observations, 289 (88.7%) had appropriate materials available to practice correct hand hygiene (water and soap in 201 [61.7%] and/or ABHR in 174 [53.4%]), while in 37 (11.3%) instances only water was available. Despite the availability of water and soap or ABHR, only 148 (51.2%) of observed students correctly performed HH. When entering school, almost all (98.8%, n = 80) students performed correct HH (Fig. 4). However, after using the restroom and before eating only 42.9% (36/84) and 25.8% (42/124) performed correct HH, respectively.

Chi-square tests of independence showed that girls (58.7%) performed correct hand hygiene more often than boys (44.4%) (X2 (1, N = 289) = 5.9217, p = 0.015) and students were more likely to perform correct HH when a HH assistant was present (75.2%) than when a HH assistant was not present (31.9%) (X2 (1, N = 289) = 53.6389, p < 0.001).

### Hand dirtiness evaluation

**Fig. 5:**
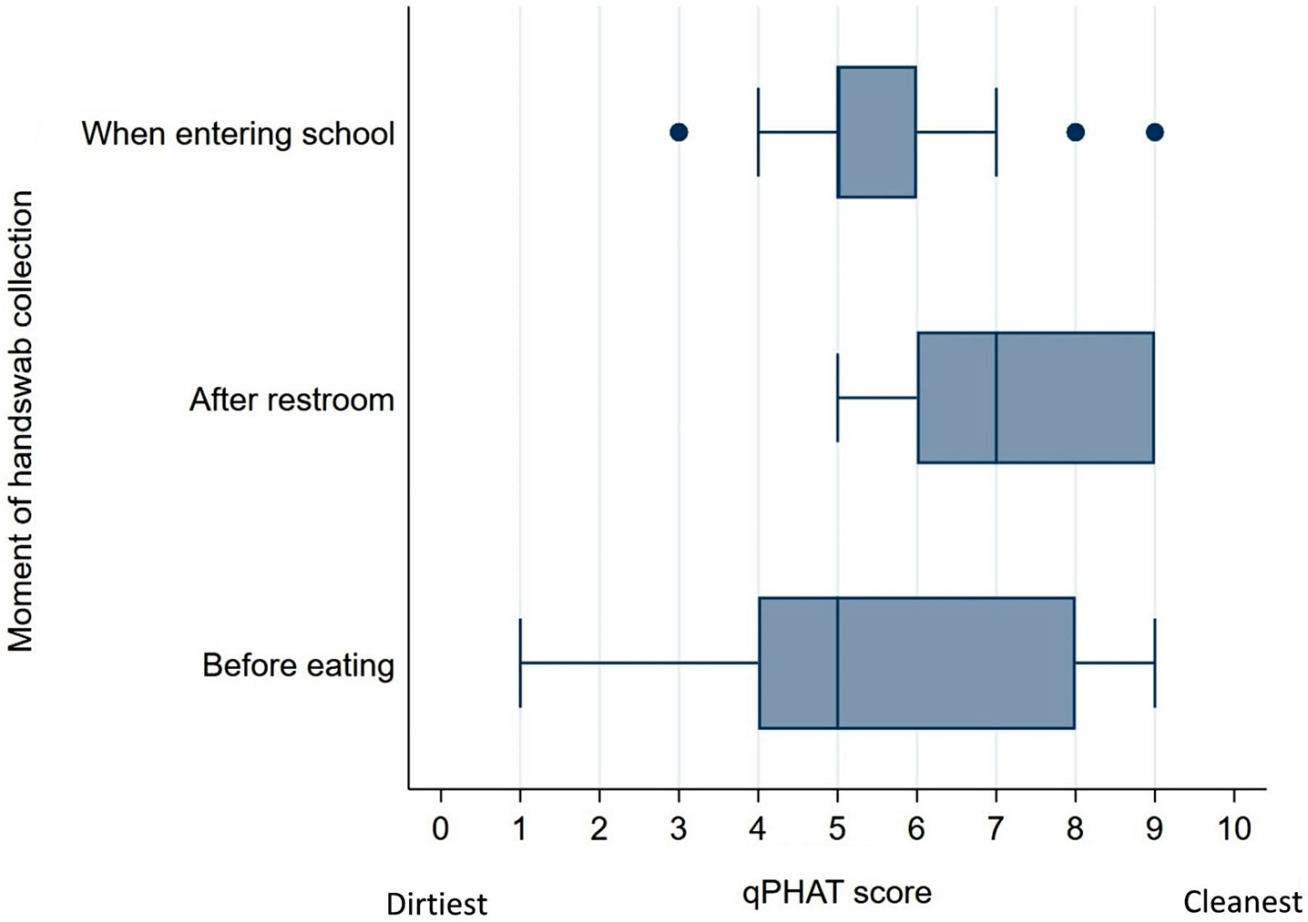
Distribution of qPHAT scores by moment of swab collection

Across all six schools, 29 (35%) swabs were collected as students were entering school, followed by 26 (32%) swabs collected after students used the restroom, and 27 (33%) swabs before students ate. Overall, the median (IQR) score was 6 [3]. Scores varied by the activity associated with the swab. Lower scores (dirtier hands) were found when students entered the school (median (IQR) = 5 (1)) and before eating (5 (4)) compared to after restroom use (7 (3)) (Fig. 5). A Kruskal Wallis test confirmed that there was a significant difference in hand dirtiness between entering the school versus hand dirtiness after using the restroom (p = 0.017) but there was no significant difference in hand dirtiness before eating and after entering the school (p = 0.6988). The median (IQR) qPHAT score among students at rural schools (n = 23 swabs) was 7 (3) compared to 5 (2) at urban schools (n = 59 swabs). The Kruskal Wallis test evidenced that this is also a significant difference where urban school students have hands with more visible dirt than students in rural schools (p < 0.05).

## Discussion

The results of this study revealed that primary school children in participating schools in Quetzaltenango had positive attitudes and good self-reported HH practices. However, students had low knowledge of critical moments for HH, as shown by the KAP surveys, and many did not practice correct HH, as indicated by observations. Additionally, there was a difference in hand dirtiness during the day, with students’ qPHAT scores varying as they arrived at school, before eating, and after restroom use.

Based on the knowledge scores alone, it appears students have high knowledge of HH in general. However, when looking at each question individually, students were unable to identify critical moments for handwashing such as after coughing or sneezing and after using the restroom. These results are similar to those of studies conducted in Uganda and Kenya where knowledge of critical times for handwashing were low prior to a curriculum-based intervention [13].

During the observations, we noted that almost all the students practiced correct hand hygiene when entering school. This could be due to the availability of ABHR at the entrances as required by the MSPAS and MINEDUC guidelines, or as a result of having a hand hygiene assistant present [6]. Schools had the assistant actively requiring students to use ABHR as they entered schools, which was associated with increased practice.

According to the observations, after using the restroom, only 42.9% of students in this study practiced correct HH. In contrast, a cross-sectional epidemiological study from schoolchildren in Zimbabwe found that out of 460 students, 60.4% of them washed their hands with soap and water after using the toilet [14]. The lack of appropriate HH practices after using the toilet may be due to a lack of knowledge of critical moments, which was evidenced in the KAP survey results (only 31.2% recognize after restroom use as a critical moment for handwashing). A similar study from Ivory Coast also discusses the importance of access to HH materials to ensure correct practice. Although not the case in our study, ensuring a constant supply of HH materials is crucial to promote positive HH behaviors [15]. A study conducted among schoolchildren in Ethiopia demonstrated that students had adequate knowledge of hygiene, but poor self-reported practices [16]. In the Ethiopia study, 99.0% of students reported washing their hands before eating but only 36.2% reported using soap. Additionally, while 76.7% of students mentioned that washing hands after restroom use is important, only 14.8% reported actually washing them after using the restroom [16]. Both our study and the study in Ethiopia highlight a disconnect between knowledge and actual practice among students.

In this study, girls were more likely to perform correct HH. Other studies have also found that there is a significant association between gender and HH practice in school age children [9, 17]. Chen et al. suggests that this is might be due to females being less likely to participate in risky behavior, (i.e. more likely to follow handwashing recommendations) and therefore wash their hands more often [17]. This is in line with a study that aimed to understand the HH practices of young adults in the context of the COVID-19 pandemic, which found that females practiced HH more often [18].

The distribution of qPHAT scores by activity show that there is a difference between hand dirtiness when entering the school compared to swabs taken after restroom use and before eating. Students’ hands were “dirtier” as they walked into school. Hand dirtiness at this time could be due to students playing or working before walking into school or due to inadequate HH practices outside of the school setting. When entering the schools, students mostly used ABHR, which only kills microorganisms but does not “remove” any debris or dirt [19]. Therefore, ABHR might not be the best HH technology at school entrances. The cleanest hands (higher scores) were after restroom use, but samples were taken regardless of whether the student washed or did not wash their hands after leaving the toilet which could explain why the scores are higher (some might have washed their hands before the samples were taken). Students’ hands were also dirty before they ate, which usually occurred after they had been in class working, painting, or writing, all of which could lead to dirtier hands.

qPHAT scores also varied between rural and urban schools, with urban schools having dirtier hands. In our sample, urban schools are larger in space, and handwashing stations are not distributed throughout the property but are centralized around the restrooms. This could make it harder for students in the urban schools in our study to wash their hands, as the HH stations are not as accessible as they are in the smaller rural schools. In contrast, studies carried out in India and Ethiopia comparing HH practices between rural and urban localities, showed that attending an urban school was a predictor for proper HH [20, 21].

The aim of this study was to establish a baseline of HH practices to inform an intervention to improve this behavior. Based on these findings, an intervention could prioritize three main aspects: first, increase awareness of critical hand hygiene moments; second, promote behavior change to establish consistent HH habits; and third, ensure the availability of adequate HH materials.

An information, education, and communication (IEC) campaign, along with environmental nudge and health messaging, could be beneficial for the participating schools as well as other educational entities in similar settings, to promote better hand hygiene behaviors [13]. A study from primary schools in the UK found that the exposure to increased infection control messaging during the influenza pandemic of 2009 played a role in the recognition of the importance of HH [22]. Data collection for this study took place during the COVID-19 pandemic so continued messaging around prevention of the disease can be leveraged to increase knowledge about how and when to practice appropriate HH at key moments. The IEC campaign should target critical moments for HH, like after using the restroom, before eating, and after coughing or sneezing. As noted previously, a HH assistant might also increase HH practices, but this may not be feasible in all settings. Environmental nudges, such as reminders to wash hands above HH stations or footpaths leading from the toilets to HH stations could provide similar cues to practice HH at key moments [23].

### Strengths and Limitations

The combination of KAP surveys, HH observations, and hand swabs is an effective mechanism to explore HH practices because it allows a thorough understanding of students’ perception on the matter as well as the identification of gaps in knowledge and behaviors.

One of the main limitations of the study was the use of convenience sampling for the selection of students that participated in the hand dirtiness evaluation and KAP surveys, which can limit representativeness and reduce external validity. Additionally, the lack of parental consent to use students´ data reduced the sample size beyond expectations. School closures due to COVID-19 governmental restrictions led to a reduction in the number of schools involved in this baseline study resulting in a small sample of schools, which may affect the external validity of the findings. Given that the qPHAT methodology is a novel metric, evidence on its validity as a reliable measure of hand dirtiness remains insufficient [11].

## Conclusions

In general, students´ knowledge, attitudes, and self-reported practices regarding HH were positive, reflecting a promising foundation for promoting health conscious behaviors. However, it is essential to acknowledge that certain aspects of HH knowledge require reinforcement. It is important for targeted interventions to improve hand hygiene practices in Guatemalan schools to be implemented collaboratively by school authorities and the Ministry of Education. School principals should ensure accessibility to appropriate materials such as water, soap, and alcohol-based hand rub that can facilitate the practice of correct hand hygiene. Simultaneously, the Ministry of Education could support efforts to include hand hygiene education as part of the formal curriculum. School-based educational campaigns could also involve students’ parents, as hand hygiene habits promoted at home can reinforce good practices at school (and vice versa). The implications of hand hygiene on students´ health extend beyond the prevention of illnesses. By promoting and fostering a culture of proper hand hygiene, Guatemalan schools can positively influence students´ overall wellbeing and their role in building healthier communities.

## Data Availability

The datasets used and/or analyzed during the current study are available from the corresponding author on reasonable request.

## List of abbreviations

HH: hand hygiene
KAP: knowledge, attitudes, and practices
qPHAT: Quantitative Personal Hygiene Assessment Tool
ABHR: alcohol-based hand rub
MINEDUC: Guatemala´s Ministry of Education
MSPAS: Guatemala´s Ministry of Health and Social Assistance
IEC: information, education and communication
